# Supplementary material for: HyLight: Strain aware assembly of low coverage metagenomes
Source: Nat Commun. 2024 Oct 7;15:8665. doi: 10.1038/s41467-024-52907-0 (PMC11458758; doi:10.1038/s41467-024-52907-0)
Supplement: Supplementary file 1 — Supplementary Information [file 41467_2024_52907_MOESM1_ESM.pdf]

HyLight: Strain aware assembly of low coverage metagenomes Supplementary Information

Xiongbin Kang[1,2], Wenhai Zhang[1], Yichen Li[3], Xiao Luo[1,*], Alexander Schönhuth[2,*]

[1] College of Biology, Hunan University, Changsha, China.
[2] Genome Data Science, Faculty of Technology, Bielefeld University, Bielefeld, Germany. [3]College of Computer Science and Electronic Engineering, Hunan University, Changsha, China

[*]To whom correspondence should be addressed.

Supplementary Tables

Supplementary Table 1. The quality of four real long reads datasets was assessed using Quast.

Raw reads	MC(%)	Indels/100kbp	Mismatches/100kbp	GF(%)	N50
Bmock12 ONT	2.33	4001.58	2659.11	93.63	22772
Bmock12 PacBio	7.55	7790.78	1851.43	87.98	8701
NWCs ONT	7.18	7991.02	5476.30	100.00	9918
NWCs PacBio	7.54	11479.15	5270.85	81.61	11725

Supplementary Table 2. The genome fraction of each individual strain in the Bmock12 data (Illumina and PacBio). Present the impact of different sequencing coverage in distinct assembly methods. Because these are the only four strains in Bmock12, their assembly is more challenging than others, so they are highlighted in bold to emphasize their importance.

Assembly	Coverage (Illumina)	Coverage (PacBio)	HyLight	MetaPlatanus	OPERA-MS
**Halomonas sp.HL-4**	**507.08**	**40.01**	**92.73**	**82.19**	**62.44**
**Halomonas sp.HL-93**	**579.87**	**45.99**	**95.57**	**98.54**	**94.92**
**Marinobacter sp.LV10R510-8**	**447.83**	**39.57**	**100.00**	**99.97**	**99.66**
**Marinobacter sp.LV10MA510-1**	**135.05**	**12.86**	**99.84**	**99.85**	**98.96**
Muricauda sp.ES.050	618.76	50.23	100.00	99.92	99.70
Psychrobacter sp.LV10R520-6	425.47	39.30	99.86	99.34	98.34
Cohaesibacter sp.ES.047	170.59	17.91	99.54	99.28	98.32
Thioclava sp.ES.032	78.32	9.49	99.56	99.47	99.32
Propionibacteriaceae bacterium	31.90	4.31	100.00	99.99	99.97
Micromonospora echinofusca	18.19	3.69	99.66	99.53	99.23
Micromonospora echinaurantiaca	14.91	3.02	99.59	99.67	99.19

Supplementary Table 3. Coverage and average nucleotide identity (ANI) of strains in the NWc data set.

Genomes	GenBank no	ANI (%)
Streptococcus_thermophilus_isolate_NWC_1_1	CP029252.1	99.99
Streptococcus_thermophilus_isolate_NWC_2_1	CP031021.1	
Lactobacillus_delbrueckii_isolate_NWC_1_2	CP029250.1	99.24
Lactobacillus_delbrueckii_isolate_NWC_2_2	CP031023.1	
Lactobacillus_helveticus_isolate_NWC_2_4	CP031018.1	98.03
Lactobacillus_helveticus_isolate_NWC_2_3	CP031016.1	

Supplementary Table 4. NWCs ONT. The genome fraction of each individual strain in the NWCs data (Illumina and ONT). Present the impact of different sequencing coverage in distinct assembly methods.

Assembly	Coverage (Illumina)	Coverage (ONT)	HyLight	OPERA-MS	Unicycler	hybridSPAdes
Streptococcus_thermophilus_isolate_NWC_1_1	56.29	84.08	99.94	98.03	50.11	83.49
Streptococcus_thermophilus_isolate_NWC_2_1	55.07	75.20	99.99	92.40	89.88	76.19
Lactobacillus_delbrueckii_isolate_NWC_1_2	39.38	25.68	96.33	90.10	92.46	75.85
Lactobacillus_delbrueckii_isolate_NWC_2_2	35.13	38.77	96.59	89.41	80.89	24.91
Lactobacillus_helveticus_isolate_NWC_2_4	17.59	221.79	98.09	93.00	82.22	63.47
Lactobacillus_helveticus_isolate_NWC_2_3	10.27	90.80	90.85	77.00	55.63	32.22

Supplementary Table 5. NWCs PacBio. The genome fraction of each individual strain in the NWCs data (Illumina and PacBio). Present the impact of different sequencing coverage in distinct assembly methods.

Assembly	Coverage (Illumina)	Coverage (PacBio)	HyLight	OPERA-MS	Unicycler	hybridSPAdes
Streptococcus_thermophilus_isolate_NWC_1_1	56.29	243.26	96.30	95.25	98.77	82.99
Streptococcus_thermophilus_isolate_NWC_2_1	55.07	190.32	93.67	83.22	89.53	69.80
Lactobacillus_delbrueckii_isolate_NWC_1_2	39.38	180.43	93.58	86.48	97.58	75.01
Lactobacillus_delbrueckii_isolate_NWC_2_2	35.13	36.19	72.76	81.37	82.84	20.96
Lactobacillus_helveticus_isolate_NWC_2_4	17.59	1.45	70.26	65.34	39.30	54.69
Lactobacillus_helveticus_isolate_NWC_2_3	10.27	2.47	41.08	40.54	12.38	37.36

Supplementary Table 6. A comparison of assembly result quality is performed between HyLight and Strainberry. Indels/100 kbp: average number of insertion or deletion errors per 100,000 aligned bases. Mismatches/100 kbp = average number of mismatch errors per 100,000 aligned bases. Genome Fraction GF reflects how much of each of the strain-specific genomes is covered by contigs. N/100 kbp denotes the average number of uncalled bases (N's) per 100,000 bases in contigs. MC = fraction of misassembled contigs.

Assembly	GF(%)	Indels/100kbp	Mismatches/100kbp	NGA50	N/100 kbp	MC(%)
3 salmonella						
HyLight	97.13	1.27	15.00	356582	0.00	0.22
Strainberry	-	-	-	-	-	-
20 strains						
HyLight	92.15	5.55	58.66	139730	0.00	0.33
Strainberry	78.43	8.48	26.26	95338	19.60	2.30
100 strains						
HyLight	93.86	11.68	55.68	163296	0.00	1.00
Strainberry	79.25	210.30	115.87	65706	68.05	4.84
210 strains						
HyLight	90.16	17.69	52.78	128015	0.00	1.63
Strainberry	78.56	418.36	102.55	81842	41.72	4.66
Bmock12 ONT						
HyLight	99.77	1.45	3.58	281944	0.00	3.59
Strainberry	67.60	705.34	264.57	688598	3.57	11.66
Bmock12 PacBio						
HyLight	98.57	5.29	19.24	123823	0.00	7.62
Strainberry	62.50	272.41	33.48	72377	21.30	22.17
NWC ONT						
HyLight	95.35	30.45	174.89	62800	0.00	9.37
Strainberry	91.69	764.14	193.84	141570	60.26	22.94
NWC PacBio						
HyLight	78.94	84.42	219.74	22388	0.00	4.27
Strainberry	43.03	246.94	111.47	-	0.65	22.22

Supplementary Table 7. Running times of different assembly approaches.

Methods	CPU time (h)	Peak Memory Usage (GB)
OPERA-MS	2.09	1.23
hybridSPAdes	5.53	3.85
HyLight	7.01	15.99
MetaPlatanus	6.93	69.26
Unicycler	53.71	6.53

Supplementary Table 8. The assembly results for the S288C yeast strain were obtained, enabling a comparison between the assembly produced using HyLight, as a hybrid approach that combines NGS and (noisy) ONT data, and assemblies of leading HiFi assemblers, generated solely from high-quality HiFi reads. Average read length pertains to long reads (ONT or HiFi) only. In the same comparison group, the best-performing results are highlighted in bold to emphasize their significance.

Assembly	HyLight (Hybrid)	Hifiasm-meta (HiFi)	MetaMDBG (HiFi)
GF (%)	**99.65**	98.56	98.16
Indels/100kbp	**41.18**	118.85	81.81
Mismatches/100kbp	**346.46**	694.54	715.85
N/100kbp	0.01	0	0
NGA50	140483	**273641**	183302
MC(%)	**36.63**	57.98	49.25
Average Read Length	21343.21	20209.19	20209.19
