# Supplementary material for: HyLight: Strain aware assembly of low coverage metagenomes
Source: Nat Commun. 2024 Oct 7;15:8665. doi: 10.1038/s41467-024-52907-0 (PMC11458758; doi:10.1038/s41467-024-52907-0)
Supplement: Supplementary file 3 — Description of Additional Supplementary Files [file 41467_2024_52907_MOESM3_ESM.pdf]

## Description of Additional Supplementary Files

**File name:** Supplementary Data 1-6

**Description:** This Excel file contains six data sets. Supplementary Data 1 provides the sample ID information for three Salmonella samples and their Average Nucleotide Identity (ANI). Supplementary Data 2 includes the sample ID information for 20 bacterial strains, detailing their species and the ANI between them. Supplementary Data 3 outlines the sample ID information for 100 bacterial strains, indicating their corresponding species and the ANI between them. Supplementary Data 4 describes the sample ID information for 210 bacterial strains, including their respective species and the ANI between them. Supplementary Data 5 lists the sample ID information for 10 Salmonella strains used as spike-ins, along with the assembly results from different software for these mixed samples. Supplementary Data 6 compares the genome fraction assembly differences among 100 bacterial strains using HyLight, MetaPlatanus, and OPERA-MS software across varying sequencing depths.
